# Enoxaparin Prevents Steroid-Related Avascular Necrosis of the Femoral Head

**DOI:** 10.1155/2014/347813

**Published:** 2014-07-02

**Authors:** Rainer Beckmann, Hayfaa Shaheen, Nisreen Kweider, Alireza Ghassemi, Athanassios Fragoulis, Benita Hermanns-Sachweh, Thomas Pufe, Mamed Kadyrov, Wolf Drescher

**Affiliations:** ^1^Department of Anatomy and Cell Biology, RWTH Aachen University, Wendlingweg 2, 52074 Aachen, Germany; ^2^Department of Oral and Maxillofacial Surgery, RWTH Aachen University, Pauwelsstraße 30, 52074 Aachen, Germany; ^3^Department of Pathology, RWTH Aachen University, Pauwelsstraße 30, 52074 Aachen, Germany; ^4^Department of Orthopedic and Trauma Surgery, RWTH Aachen University, Pauwelsstraße 30, 52074 Aachen, Germany; ^5^Department of Orthopaedic and Spine Surgery, AGAPLESION EV. BATHILDISKRANKENHAUS gemeinnützige GmbH, Maulbeerallee 4, 31812 Bad Pyrmont, Germany

## Abstract

Nontraumatic osteonecrosis of the femoral head is still a challenging problem in orthopedic surgery. It is responsible for 10% of the 500,000 hip replacement surgeries in the USA and affects relatively young, active patients in particular. Main reasons for nontraumatic osteonecrosis are glucocorticoid use, alcoholism, thrombophilia, and hypofibrinolysis (Glueck et al., 1997; Orth and Anagnostakos, 2013). One pathomechanism of steroid-induced osteonecrosis is thought to be impaired blood flow to the femoral head caused by increased thrombus formation and vasoconstriction. To investigate the preventive effect of enoxaparin on steroid-related osteonecrosis, we used male New Zealand white rabbits. Osteonecrosis was induced by methylprednisolone-injection (1 × 20 mg/kg body weight). Control animals were treated with phosphate-buffered saline. Treatment consisted of an injection of 11.7 mg/kg body weight of enoxaparin per day (Clexane) in addition to methylprednisolone. Four weeks after methylprednisolone-injection the animals were sacrificed. Histology (hematoxylin-eosin and Ladewig staining) was performed, and empty lacunae and histological signs of osteonecrosis were quantified. Histomorphometry revealed a significant increase in empty lacunae and necrotic changed osteocytes in glucocorticoid-treated animals as compared with the glucocorticoid- and Clexane-treated animals and with the control group. No significant difference was detected between the glucocorticoid and Clexane group and the control group. This finding suggests that cotreatment with enoxaparin has the potential to prevent steroid-associated osteonecrosis.

## 1. Introduction

Nontraumatic osteonecrosis of the femoral head remains a challenge to orthopedic surgeons. If untreated, it leads in 80% of cases to complete collapse of the femoral head [1]. The annual incidence of osteonecrosis in the USA is 50 000 patients per year, most commonly in young, active patients with an average age of 38 [2]. The etiology of nontraumatic osteonecrosis is still not fully elucidated. Osteonecrosis has a multifactorial etiology. Beside glucocorticoid therapy and alcoholism, hematological diseases like thrombophilia, hypofibrinolysis, and sickle-cell anemia and metabolic diseases such as Gaucher's disease are associated with osteonecrosis [1–3]. Glucocorticoids also directly affect osteocytes. They induce osteocyte apoptosis, the first histological sign of osteonecrosis [1, 2]. Osteocytes are important for the orchestration of the bone cells. The death of these cells leads to an impairment of bone remodeling and finally to a loss of structural integrity. Another much-discussed theory of osteonecrosis etiology is ischemia with subsequent damage to the affected tissue. Glucocorticoid therapy affects the blood supply. Glucocorticoids induce fat-cell hypertrophy in the bone marrow and increase intraosseous pressure [4]. Glucocorticoids also may directly affect blood supply by increasing vasoconstriction. This constellation is further enhanced by hyperlipidemia, hypercoagulation, and hypofibrinolysis of the circulatory system all of which are caused by glucocorticoid use [5].

The pathogenesis of glucocorticoid-induced osteonecrosis may involve intravascular thrombotic occlusion, extravascular lipid deposition by fat emboli, increased intraosseous lipocyte size, or any combination of these. This can increase bone-marrow pressure and exacerbate intraosseous circulatory disturbances, resulting in insufficient blood supply and finally in necrosis [6]. Enoxaparin is an established drug for thrombosis prevention. Glueck et al. could arrest the progression of Ficat I-II osteonecrosis in patients with primary osteonecrosis by enoxaparin administration in a human pilot study [7]. Also in a rat model of mechanical induced osteonecrosis by cutting the ligamentum teres and incessing the periosteum showed enoxaparin treatment positive effects. The treatment led to restoration of the necrotic area within 4 weeks [8, 9]. Because of the positive effect of enoxaparin in inhibition of the progression of osteonecrosis and the reparative capacity in a mechanical induced osteonecrosis model, we investigated the preventive potential of enoxaparin cotreatment during high dose glucocorticoid therapies.

## 2. Materials and Methods 

Osteonecrosis was induced in New Zealand White rabbits (male; 3–4.5 kg body weight) by injecting once 20 mg/kg body weight methylprednisolone i.m. (GC group; *n* = 6), and control animals (*n* = 6) were treated with phosphate-buffered saline (PBS). The therapy group (GC + Clexane; *n* = 6) received 11,7 mg/kg body weight per day enoxaparin sodium starting with the methylprednisolone administration. The animals were sacrificed four weeks after methylprednisolone injection. For histology, tissue samples were fixed in 3% paraformaldehyde, decalcified in 10% EDTA-Tris buffer, and embedded in paraffin. Hematoxylin and eosin and Ladewig stains were performed on 5 *μ*m thick slides. This experiment complied with the German Law on Animal Experiments and was approved by regional committee of animal experimentation ethics (Ministerium für Energiewende, Landwirtschaft, Umwelt und ländliche Räume des Landes Schleswig-Holstein: V742-72241.121-39 (70-9/04)).

### 2.1. Quantification of Empty Osteocyte Lacunae

Measurements were performed on a stereological workstation (Olympus CAST grid; Olympus, Albertslund, Denmark; Olympus BX 50 microscope), and sections were visualized with a CCD color video camera (600 × 800 pixels; JAI, Glostrup, Denmark) on a 17′′ monitor. The area of interest, covering the whole femoral head, was delineated on serial hematoxylin and eosin stained sections at low magnification (lens: ×1.25; PlanApo). A computer program using systemic random sampling selected 5 fields within the area of interest (in the middle of the femoral head). Fields were evaluated per section (lens: ×40; UPlanApo, NA = 0.70) by superimposing a grid of 16 crosses and counting the number of empty lacunae and the area of necrotic changes per 1 mm^2^. Two slides of the right femoral head per animal were evaluated. Three blinded investigators performed counting of empty osteocyte lacunae and the number of necrotic changed osteocytes (cells degenerating: pyknosis, karyorrhexis, karyolysis).

### 2.2. Statistical Analysis

Numerical densities of empty lacunae and areas exhibiting necrotic changes were calculated. The mean and SEM were calculated for every single section and for every group prior to calculation of the mean and SEM for each group and each investigated variable. The Bartlett test was used to check for equal variances. BoxCox transformation was performed if necessary to achieve homoscedasticity. Normal distribution was tested with the Shapiro-Wilk test. Parametric data were analyzed with one-way ANOVA followed by Tukey *t*-test. Statistical significance was established at *P* < 0.05. Statistical analysis was performed using Graph Pad Prism 5 and JMP 10.

## 3. Results

**Figure 1:**
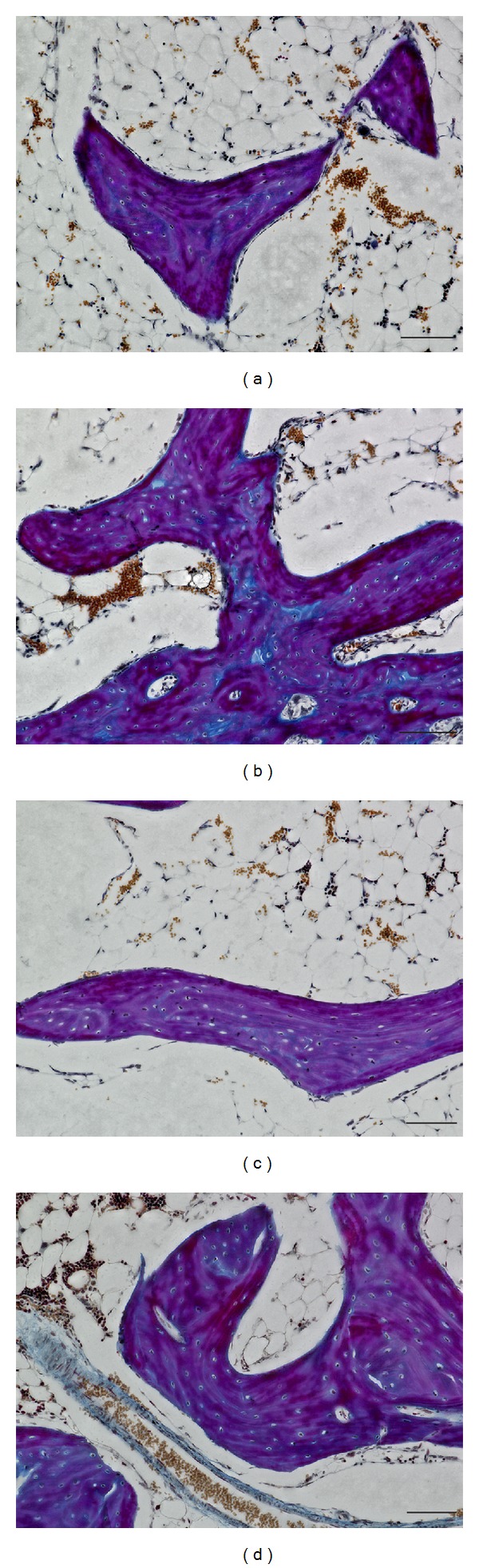
Ladewig stained sagittal sections of the femoral head. Methylprednisolone treatment induces osteonecrosis demonstrated by an increase of empty osteocyte lacunae (c) in comparison to PBS treated control animals (a and b). Enoxaparin cotreatment leads to a decrease of empty osteocyte lacunae and adipocytes (d). Bar represents 100 *μ*m.

**Figure 2:**
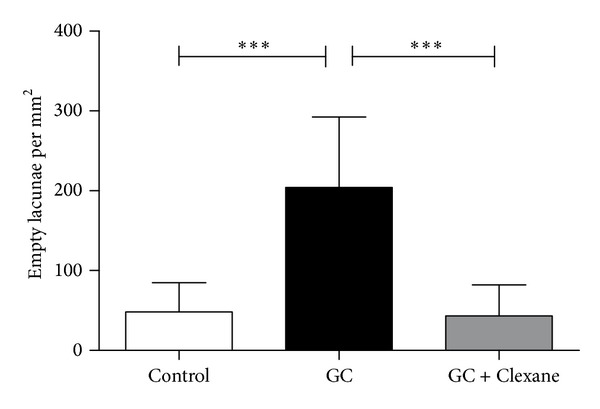
Enoxaparin treatment decreases glucocorticoid-induced osteonecrosis. The application of enoxaparin with methylprednisolone reduces significantly the amount of empty osteocyte lacunae per mm^2^ in the femoral head of rabbits due to methylprednisolone treatment to control levels. The graph represents the mean score with SEM of empty lacunae. Statistical analysis was performed with one-way ANOVA followed by Bonferroni's multiple comparison test (control versus GC, control versus GC + Clexane, and GC versus GC + Clexane). (***P < 0.0001).

**Figure 3:**
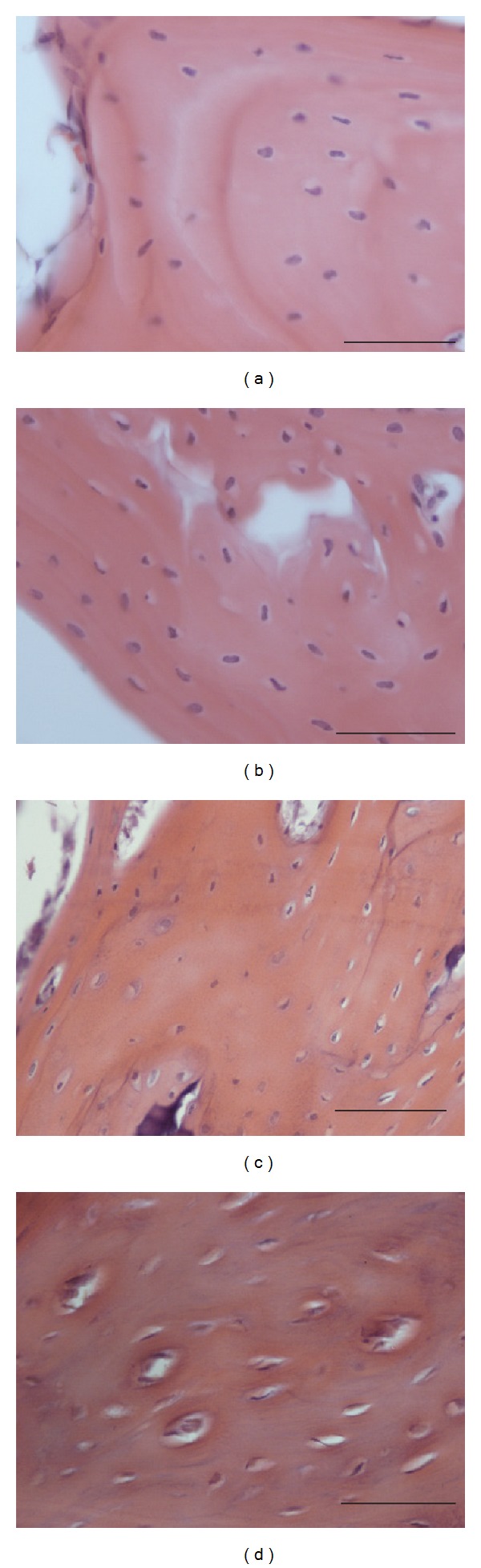
Enoxaparin treatment prevents necrotic changes of osteocytes. Methylprednisolone treatment induced osteonecrosis demonstrated by necrotic changes of osteocytes (c + d) in comparison to PBS treated control animals (a). Enoxaparin cotreatment leads to a decrease of necrotic changed osteocytes (b). Bar represents 50 *μ*m.

**Figure 4:**
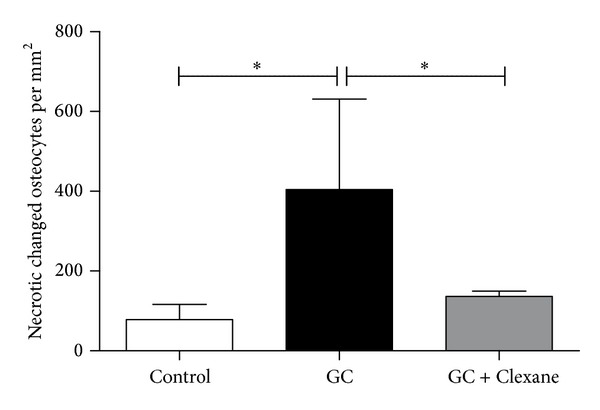
Enoxaparin treatment decreases glucocorticoid-induced necrotic changes of osteocytes. The application of enoxaparin with methylprednisolone reduces significantly the amount necrotic changed osteocytes per mm^2^ in the femoral head of rabbits due to methylprednisolone treatment to control levels. The graph represents the mean score with SEM of empty lacunae. Statistical analysis was performed with one-way ANOVA followed by Bonferroni's multiple comparison test (control versus GC, control versus GC + Clexane, and GC versus GC + Clexane). (*P < 0.05).

Ladewig staining revealed normal bone-tissue structure in the control group (Figures 1(a) and 1(b)) and a strong abnormal increase in empty osteocyte lacunae in the glucocorticoid-treated group (Figure 1(c)). In the group treated with enoxaparin the number of empty osteocyte lacunae returned to control level (Figure 1(d)). The bone-lining cells appeared to have less number of pyknotic changed nuclei in the enoxaparin-treated group compared to the GC group (Figure 1(d)). Also the number of osteonecrotic changed osteocytes increases after glucocorticoid treatment (Figure 2). The cotreatment with enoxaparin reduced the necrotic signs in osteocytes (Figure 2). Histomorphometry revealed a significant increase in empty lacunae and necrotic changed osteocytes in glucocorticoid-treated (GC-treated) animals as compared both with controls and with animals treated with glucocorticoid and enoxaparin (control versus GC, control versus GC + Clexane and GC versus GC + Clexane; *P* < 0.0001). No significant differences in the number of empty lacunae nor necrotic changed osteocytes were detected between the glucocorticoid and enoxaparin cotreated group (GC + Clexane) and the control group (Figures 2, 3 and 4).

## 4. Discussion

The literature contains several rabbit models of steroid-induced osteonecrosis [6, 10]. We chose, from among these, the osteonecrosis model established by Yamamoto et al. [11]. We used this model to address the question whether the anticoagulant enoxaparin could be used as a preventive therapy against glucocorticoid-induced osteonecrosis [5, 7]. In the current study, administration of the glucocorticoid methylprednisolone leads to osteonecrosis in the femoral head, as demonstrated by the increased number of empty osteocyte lacunae and osteonecrotic changed osteocytes, which are pathological features of osteonecrosis. The additional treatment with enoxaparin results in a subsequent decrease in the number of necrotic changed osteocytes and empty lacunae as compared with the GC animals. This suggests that cotreatment with enoxaparin may have a preventive effect against steroid-associated osteonecrosis. The association between corticosteroid therapy and osteonecrosis has been well established since 1957, when the first case report of vascular lesions from glucocorticoid therapy in patients with rheumatoid arthritis was published [12]. The femoral head, because of its nonredundant blood supply, is vulnerable to ischemic damage caused by capillary occlusions. One much-discussed pathomechanism of osteonecrosis of the femoral head is ischemia. Glucocorticoids lead to vasoconstriction. Drescher and colleagues [9, 13] were previously able to demonstrate increased vasoconstriction and coagulability following high-dose glucocorticoid application in a swine model—possibly leading to reduced blood supply and enhanced capillary occlusion causing ischemia. Glucocorticoids are known to suppress the production of vasodilators such as prostacyclin and nitric oxide [14]. But glucocorticoids also modulate the vascular response to vasoregulators. In many species, such as rats, rabbits, and humans, an enhanced vasoconstrictive response to catecholamines and endothelin has been observed, while the response to bradykinin, a vasodilator, was reduced [12, 15]. Beside the general reduced oxygen and nutrient supply caused by vasoconstriction, the literature also discusses heritable or acquired risk factors for femoral head osteonecrosis related to hypercoagulability, hemoglobinopathies, steroids, angiogenesis, and oxidative stress [16]. Dysregulated coagulation parameters play a substantial role in the etiology of osteonecrosis reviewed by Orth and Anagnostakos [3]. Glueck et al. could demonstrate thrombophilia and hypofibrinolysis in patients with idiopathic and GC induced secondary osteonecrosis [17]. Mutations of the factor V Leiden and prothrombin genes could be observed in patients with idiopathic osteonecrosis and secondary osteonecrosis [18, 19]. Several studies have demonstrated the involvement of plasminogen activator inhibitor-1 (PIA-1). PIA-1 suppresses the generation of plasmin, leading to hypofibrinolysis. Mutation in the PIA-1 gene was identified as a risk factor of glucocorticoid-induced osteonecrosis [14, 20]. Kerachian et al. could observe an increased PIA-1 expression in prednisone-induced osteonecrosis model in Wistar rats by Affymetrix analysis [21].

The pathomechanism of osteonecrosis also involves other factors of coagulation regulation. Wu et al. [22], in a proteomic study, were able to show a lower level of antithrombin III, an inhibitor of the blood coagulation, in the serum of patients with osteonecrosis. M. L. te Winkel et al. [23] were also able to show a decrease in antithrombin serum level, as well as a decrease in the level of protein S, a cofactor of protein C, which acts as an anticoagulative. The expression of these anticoagulative proteins differs significantly in patients who developed osteonecrosis during dexamethasone treatment as compared with patients who did not develop osteonecrosis. M. L. te Winkel et al. [23] also showed increased expression of factor X after 3 weeks of dexamethasone treatment contributing to development of symptomatic osteonecrosis. Cenni et al. [24] showed also reduced inhibition of coagulation due to reduced protein C activity, in combination with hypercoagulation due to increased D-dimer formation in glucocorticoid-induced osteonecrosis in a group of 18 patients. Platelet aggregation is also modulated by glucocorticoids. Sebaldt et al. [25] found that patients with osteonecrosis showed inhibited synthesis of prostacyclin (PGI_2_), a potent inhibitor of platelet aggregation.

Intravascular coagulation is also caused by fat emboli, a complication after glucocorticoid therapy. Fat emboli deposits in vessels and sinusoids may indirectly induce coagulation by activating the complement pathway and causing deposition of immune complex [26].

Glucocorticoid treatment influences the coagulation and thrombus formation at several points. The increased coagulation in combination with the induced vasoconstriction by glucocorticoids enhances the risk of capillary occlusion followed by necrosis.

In this study, we investigate the effect of heparin derivate enoxaparin treatment as a preventive therapy option for use in conjunction with glucocorticoid therapies. Enoxaparin is a commonly used drug for thrombosis prevention. The main action of heparin is to increase inhibition of the serine protease factor Xa, the critical component connecting the intrinsic and extrinsic activations of the coagulation pathway. Heparin binds to antithrombin III and induces a conformation change. The ability of this complex to render factor Xa inactive thereby undergoes a 300-fold increase as compared to antithrombin III by itself. A second mechanism is the binding of the heparin-antithrombin III-complex to thrombin, which leads to the inactivation of thrombin. Thrombin is required to convert soluble fibrinogen into an insoluble fibrin clot (reviewed in [27]).

In clinical trials, enoxaparin has proved more effective than other heparins in managing acute coronary syndromes (ACS) [22, 23]. Low-molecular-weight heparins (LMWHs) are used as anticoagulant and antithrombotic drugs. They are more effective against both venous and arterial thrombosis than unfractionated heparin [28]. By comparison, LMWHs have a lower affinity to plasma proteins and endothelial cells, a higher affinity to factor Xa, a greater capacity to release tissue factor pathway inhibitor from endothelial cells, a more predictable dose-response relationship, and a longer plasma half-life with dose-independent clearance kinetics. They are also relatively resistant to neutralization by platelet factor 4 [28]. It is now widely accepted that LMWHs have individual biochemical and pharmacological profiles [25, 26].

Several clinical studies were performed to access enoxaparin as a therapy option to stop the progression of osteonecrosis. Glueck et al. could arrest the progression of osteonecrosis of Ficat stages 1 and 2 by enoxaparin therapy in 19 of 20 patients with thrombophilic-hypofibrinolytic disorders over an observation period of 24-month follow-up, and only 20% of Ficat stages 1 and 2 hips of patients with secondary osteonecrosis did not progress further [7]. The patients of this study have already a manifested osteonecrosis. Our study demonstrates a preventive effect of enoxaparin treatment when simultaneously administrated with high dose glucocorticoid therapies. We were able to demonstrate significant reduced signs of early osteonecrosis. The simultaneous administration of Enoxaparin to high dose GC reduces the number of necrotic changed osteocytes and empty osteocyte lacunae. The use of enoxaparin in a chirurgical induced osteonecrosis rat model demonstrated an improvement of quantities of necrotic and newly formed bone compared to control group after 1 month [7].

Future clinical studies are needed to clarify the extent to which patients receiving high doses of GC can benefit from cotreatment with enoxaparin to prevent osteonecrosis. One salient area of relevance for such study will be in organ transplantation, where, due to progress in terms of immunosuppressive treatment and surgical techniques, the survival rate of transplant patients has increased as well, approaching 90% of patients 5 years or more after kidney transplant [29]. In spite of these advances, a number of complications may still affect the success of organ substitution. Of these, bone disease is one of the most frequently reported [29]. Patients undergoing immunosuppressive therapy with steroids may spare damage to the femoral head simply by cotreatment with enoxaparin.

## 5. Conclusion

Enoxaparin cotreatment has the potential to inhibit the osteocyte necrosis, a severe side effect of high dose glucocorticoid therapies.
