# Corrigendum to “Enoxaparin Prevents Steroid-Related Avascular Necrosis of the Femoral Head”

**DOI:** 10.1155/2015/264241

**Published:** 2015-08-26

**Authors:** Rainer Beckmann, Hayfaa Shaheen, Nisreen Kweider, Alireza Ghassemi, Athanassios Fragoulis, Benita Hermanns-Sachweh, Thomas Pufe, Mamed Kadyrov, Wolf Drescher

**Affiliations:** ^1^Department of Anatomy and Cell Biology, RWTH Aachen University, Wendlingweg 2, 52074 Aachen, Germany; ^2^Department of Oral and Maxillofacial Surgery, RWTH Aachen University, Pauwelsstraße 30, 52074 Aachen, Germany; ^3^Department of Pathology, RWTH Aachen University, Pauwelsstraße 30, 52074 Aachen, Germany; ^4^Department of Orthopedic and Trauma Surgery, RWTH Aachen University, Pauwelsstraße 30, 52074 Aachen, Germany; ^5^Department of Orthopaedic and Spine Surgery, AGAPLESION EV. BATHILDISKRANKENHAUS gemeinnützige GmbH, Maulbeerallee 4, 31812 Bad Pyrmont, Germany

In the paper titled “Enoxaparin Prevents Steroid-Related Avascular Necrosis of the Femoral Head” [1], Rainer Beckmann and Hayfaa Shaheen equally contributed to this work; Mamed Kadyrov and Wolf Drescher contributed equally as corresponding authors.
